# Using larval barcoding to estimate stomatopod species richness at Lizard Island, Australia for conservation monitoring

**DOI:** 10.1038/s41598-020-67696-x

**Published:** 2020-07-03

**Authors:** Sitara Palecanda, Kathryn D. Feller, Megan L. Porter

**Affiliations:** 1 Department of Biology, University of Hawai‘i at Mānoa, Honolulu, HI USA; 2 Department of Biological Sciences, University of Bristol, Bristol, UK; 3 Present Address: Department of Biological Sciences, Union College, Schenectady, NY USA

**Keywords:** Conservation biology, Genetics

## Abstract

Stomatopods (Crustacea, Stomatopoda) are well studied for their aggressive behavior and unique visual system as well as their commercial importance in Asian and European countries. Like many crustaceans, stomatopods undergo indirect development, passing though several larval stages before reaching maturity. Adult stomatopods can be difficult to catch due to their inaccessible habitats and cryptic coloration. By sampling larvae from the planktonic community, less effort is required to obtain accurate measures of species richness within a region. Stomatopod larvae were collected between 2006 and 2015 from the waters around the Lizard Island reef platform in Eastern Australia. Cytochrome oxidase I (COI) mitochondrial DNA sequences were generated from each larval sample and compared to a database of COI sequences tied to adult specimens. Of the 20 species collected from Lizard Island as adults which have COI data available, 18 species were identified from larval sampling. One additional species identified from larval samples, *Busquilla plantei*, was previously unknown from Lizard Island. Nine larval OTUs were found not to match any published adult sequences. Sampling larval stomatopod populations provides a comparable picture of the adult population to benthic sampling methods and may include species richness beyond what is measurable by sampling adult populations.

## Introduction

For most marine genera there exist geographic locations of very high diversity. These areas are determined by ecological factors such as water temperature, food availability, and proper habitat^1,2^. Management and conservation efforts will naturally focus on regions where species richness is greatest for groups of particular interest, but first those areas must be identified. Marine sampling methods are often more accurate for vertebrates than invertebrates, which often have life histories that are less well understood and can be found in habitats that are more difficult to sample^3–5^. In particular, biases towards groups that are easy to identify morphologically in the field present an ongoing issue in complex coral reef ecosystems^6^. In this paper we argue that sampling from the plankton provides an accurate method of estimating coral reef species richness and has the potential to be more efficient and cost-effective than attempting to catch adult representatives of each species in a group. We use the marine crustacean order Stomatopoda to present a case study of species richness estimates from planktonic sampling in the Lizard Island reef platform (Queensland, Australia). Coral reef habitats similar to and including Lizard Island have been the location of extensive research on stomatopods^7–11^. Lizard Island has the additional advantage of being regularly sampled for stomatopod species for over 30 years, providing a solid baseline of species richness in the group [^12^, R. Caldwell, personal communication].

Cytochrome oxidase I (COI) was used to perform DNA barcoding of larval stomatopods for identification and quantification of stomatopod species richness at Lizard Island. Morphological identification of stomatopod larvae is constrained by the existence of multiple larval stages for each species, some of which bear little resemblance to each other. COI barcoding is an established tool for effectively circumventing this issue with stomatopod larval identification^13–15^. By comparing COI results between larval and adult sampling profiles we can generate an accurate picture of how sampling from the plankton compares to traditional sampling techniques (i.e. catching adult animals from the benthos). In this context we are not using barcoding to definitively prove the existence of new species, but rather to generate a fast and inexpensive estimate of stomatopod species richness in a localized region. Such an efficient tool for estimating species richness may prove useful in a conservation context^16,17^.

Presently, several studies have utilized DNA barcoding as a means of identifying larval stomatopods for experimental^11,18^, taxonomic^19,20^, and diversity or distribution contexts^13–15,21^. In their work in the South China Sea, Tang et al.^15^ verified both COI and 16S as accurate genetic markers for identifying both local and commercially important stomatopod species, lending credence to earlier work by Barber and Boyce^13^ that used larval COI sequences to estimate stomatopod species richness among different geographical locations in the Indo-Pacific. In most marine species richness or diversity studies that include stomatopods, molecular data is solely derived from adult animals^22, 23^. While the Indo-Pacific is comparatively well sampled for both adult and larval stomatopods^12,13,23^, no present work compares adult versus larval sampling methods within this region. Without such a comparison, we cannot be sure of the strengths or weaknesses surrounding each method in the context of quantifying species diversity.

Another important way in which larval sampling can enhance conservation efforts is by providing a source of data to assess the effects of environmental changes, both natural and anthropogenic. Damage to a population and subsequent recovery can be difficult to assess in marine ecosystems without a comprehensive sampling regime that provides data over time and establishes a baseline for diversity^24,25^. Larval populations could also give us information to assess population connectivity, making it a valuable resource for strategical planning. A striking example comes from the Island of Krakatau, where a volcanic eruption created a situation where previously inhabited reef habitat had to be completely recolonized. Studies on stomatopod diversity at the new Krakatau site give us clues about larval connectivity and how close together marine protected areas need to be in order to be effective^21^. Baseline data can help to make studies like the one conducted in Krakatau even more effective at determining directions for conservation.

In 2016 the Great Barrier Reef around Lizard Island suffered one of the worst coral bleaching events in its history. Data collected before this time provides another resource in assessing the damage to coral reefs and the communities they hold. Sampling species richness from the plankton, in conjunction with previous adult sampling that provides a strong baseline for species richness estimates, can be used to make assessments of damage to Lizard Island reef habitats after large impacts and may provide insights on dealing with similar situations elsewhere.

## Methods

### Specimen collection

Sampling was conducted at the Lizard Island Research Station, off the Eastern coast of Queensland Australia, at several locations within the Lizard Island reef platform (permit numbers G06/17,935.1; G09/31,676.1; G12/35,464.1; G16/38,036.1). Larval samples were taken between May and September each year from 2009–2015. All collections took place after sunset to take advantage of the positively phototactic behavior exhibited in pelagic larval stomatopods^26^. Shallow water collections (~ 1 m depth) took place while wading near shore. An underwater light source was used to attract larvae which were then captured using hand-held dip nets. Collections on scuba (3 m–18 m depth) were performed using the same light source and larvae were collected in gallon size resealable bags. In both cases larvae were transported back to the laboratory while alive and sorted within 24 h of capture. Stomatopod larvae were maintained, either individually or with presumed conspecifics from the same collection event, in plastic cups at ambient laboratory temperature (~ 26 °C) in seawater. Within 48 h of collection stomatopod larvae were fixed in 70–95% ethanol.

Larval sampling efforts varied by year in terms of time spent in the water (10 min.–60 min.), time of collection (8 pm–10 pm), moon phase, and sampling type. A list of adult species documented from Lizard Island habitats was compiled from the Australian Museum collection database as well as personal communication [R. Caldwell; T. Cronin]. Specimens of adult stomatopods used for genetic work were collected at Lizard Island starting in 2009.

### Larval COI barcoding

DNA was extracted from whole larvae using either the NucleoSpin Tissue XS (Machery-Nagel) or the DNeasy Blood and Tissue (QIAGEN) DNA isolation kits according to the manufacturer’s instructions. Extracted samples were quantified using a Nanodrop Nd-1000 spectrophotometer and quantified DNA was used for PCR amplification of the mitochondrial cytochrome oxidase I (COI) gene. The mitochondrial COI region was targeted using degenerate primers designed for stomatopod COI genes (Forward 5′ ACG CGA CGA TGA TTA TTY TCT AC 3′; Reverse 5′ GCT CGR GTR TCI ACR TCY AT 3′) and a PCR protocol modified from Feller et al.^20^. PCR mixes were conducted with a total volume of 25 µl containing 0.2 mM of each primer, 0.2 mM dNTPs (New England Biolabs), 0.1 ng/ µl BSA, 1.25 µl DMSO, 1 unit of Hotmaster taq buffer, and 30 ng of DNA. The cycling parameters of each PCR were a single 2 min incubation at 95 °C; 40 to 50 cycles of 1 min 95 °C denaturing, 1 min 47 °C annealing, and 1.5 min 70 °C elongation; and a final elongation of 10 min at 72 °C. PCR amplicons were cleaned using EXO-SAP-IT (Thermo Fisher) and sequenced using Sanger sequencing performed by either Genewiz Inc. (Germantown, Maryland) or the Advanced Studies in Genomics, Proteomics and Bioinformatics facility at the University of Hawai’i at Mānoa (Honolulu, Hawai’i). All products were sequenced in both directions.

Trimming and editing of ambiguous base pairs, and assembly of sequence contigs was completed in Geneious R10^27^. Larval sequences from Lizard Island were verified for taxonomy (Stomatopoda) and gene identity (COI) using NCBI’s Basic Local Alignment Search Tool (BLAST). A total of 306 clean COI sequences of at least 600 base pairs were obtained.

### Genetic Identification using phylogenetic reconstruction

A total of 215 reference stomatopod COI sequences were downloaded from NCBI including representatives of 27 of 32 species documented from Lizard Island. The five remaining Lizard Island species: *Gonodactylellus micronesicus, Gonodactylellus kume, Acanthosquilla derijardi*, *Lysiosquillina sulcata*, and *Lysiosquilla suthersi*, were not included in our dataset, since COI sequences were not available. Individuals published as *Chorisquilla spinosissima* (AF205254) and *Chorisquilla hystrix* (HM138777) were found to have COI sequence similarity over 98% possibly indicating misidentification or an area where taxonomic revision is necessary. Based on published range information, individuals captured at Lizard Island were classified as *C. hystrix*^12^. Sequences from three morphologically identified adult *Pullosquilla litoralis* individuals collected in Mo’orea, French Polynesia (MT169578, MT169579, and MT169580) and one morphologically identified *Alachosquilla vicina* individual collected at Lizard Island (MT169595) were generated using the protocols described above.

Of the 82 reference sequences from stomatopod species known to exist at Lizard Island, 53 sequences (representing 20 species) were from animals collected at Lizard Island. A further three sequences were from collections in Australia, and the remaining 26 sequences were from varying locations throughout the Indo and South Pacific.

Sequences were compiled into a MAFFT alignment using the software Geneious R10^27^. Sequences were translated to check for the absence of very divergent amino acid sequences and stop codons to verify that pseudogene DNA had not been amplified^23^. The beginning and end of the alignment were trimmed until 70% of sequences had a base call at that position. After trimming, the remaining sequences ranged between 497 and 765 base pairs in length. Single base pair insertions were found in three published sequences. Because we did not have access to chromatograms for verification, these nucleotides were assumed to be the result of sequencing error and were removed. We verified that tree topology was not changed by the exclusion of these base pairs by constructing trees from alignments with and without the gaps created by single base pair insertions. A preliminary tree was created using the 215 COI reference sequences mined from NCBI, four newly generated reference sequences from *Pullosquilla litoralis* and *Alachosquilla vicina* individuals, and 306 larval sequences collected at Lizard Island.

A second tree was created which included larval sequences as well as adult reference sequences of species recorded from Lizard Island. A reference sequence from *Busquilla plantei*, a species not presently recorded from Lizard Island collections, was also included in this tree based on its < 5% divergence from an unknown larval sequence collected from Lizard Island. This tree was used in all further analysis. An additional tree containing only sequences within the superfamily Gonodactyloidea was created for easier visualization.

Maximum likelihood trees were constructed using RAxML on the CIPRES platform using default input parameters^28,29^. Two COI sequences from *Hemisquilla californiensis* were used to root all trees due to the basal position of *Hemisquilla* in published stomatopod phylogenies^10,30^. To our knowledge no members of the genus *Hemisquilla* have previously been found at Lizard Island and the only known Australian member of the genus has a range which ends several hundred miles to the south^12^. Trees were visualized in FigTree (v.1.4.3)^31^.

### Designation of operational taxonomic units (OTUs) and putative species

Using the reconstructed phylogeny, maximum and minimum sequence divergence within each clade was calculated using Geneious R10^27^. Sequences were designated as being from the same species OTU when they were within 5% divergence of a sequence published as that species. A 5% cut off was chosen because it is a genetic distance at which something can unambiguously be called a different species following the criteria of Barber and Boyce^13^. OTUs which consisted of a single unknown larval sequence were disregarded.

An additional analysis to determine putative species clusters within our dataset without a pre-determined cut-off was carried out using the Poisson tree process (PTP) method^32^. This method uses branch lengths from our previously constructed tree to estimate the expected number of nucleotide substitutions per site between branching events. The number of substitutions between species is estimated by the model to be higher than the amount of substitutions within species. The model is then able to implement two classes of Poisson processes, one which describes speciation, and another which describes branching within species, to search the tree for transition points between what would be considered inter- and intra-specific branching. Species delimitations were calculated within a maximum likelihood framework. This analysis was conducted using the bPTP web server (https://www.exelixis-lab.org/software.html).

Automatic Barcode Gap Discovery (ABGD) analysis was also used to estimate species richness in this dataset. ABGD identifies potential species boundaries by analyzing differences between intraspecific and interspecific variation within a dataset and is not influenced by phylogenetic tree reconstruction^33^. We used the publicly available online platform (https://bioinfo.mnhn.fr/abi/public/abgd/abgdweb.html) to run this analysis using both the Jukes-Cantor (JC69) and Kimura (K80) distance matrices. We began our analysis using default parameters (Pmin = 0.001, Pmax = 0.1, 10 steps, 20 Nb bins, and relative gap width = 1.5). We then refined our Pmax value to 0.03, the point after which recursive partitions became the same as the initial partition. It is important to note that ABGD analysis is most effective when the number of sequences per species is above three to five^33^. Though our dataset does not conform to this guideline we believe this analysis can still provide us with additional information.

## Results

**Figure 1:**
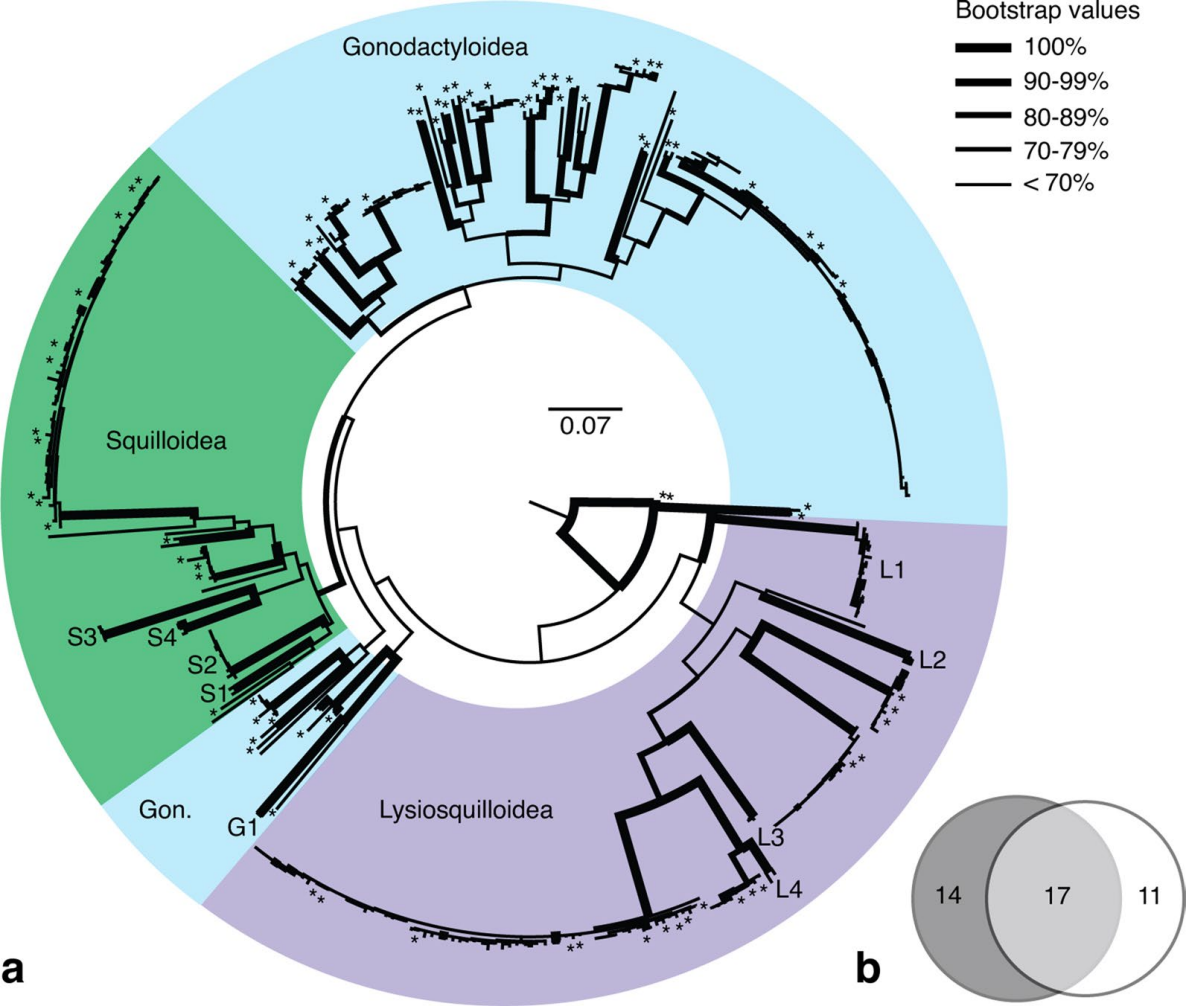
Larval and adult sequences from Lizard Island. (**a**) COI gene tree of stomatopod species from Lizard Island. The Gonodactyloidea are rendered paraphyletic. (*) symbols represent reference sequences obtained from NCBI or sequenced from morphologically identified samples. Clades containing only larval sequences are labeled by first letter of superfamily (e.g. G1, L1-4, and S1-4); all other clades contain a reference sequence. Branch thickness represents bootstrap value at each node. (**b**) Venn diagram of species morphologically or genetically identified as adults (dark circle) and as larvae (light circle) including potential new species based on our OTU designations.

Using our pre-determined 5% cut off, 252 of 306 larval samples obtained during this study from the Lizard Island reef platform were identified to species. Of the 32 species of stomatopod previously morphologically identified from the Lizard Island reef platform 20 species are represented by COI sequences from morphologically identified adults and 18 have been genetically identified from larvae (Supplementary Table S1). An additional nine OTUs were identified using 47 larval sequences, possibly representing undescribed stomatopod diversity that was missed through only sampling adults. One of these clades (G1) is within the gonodactyloids, with the remaining clades split between the lysiosquilloids (L1–L4) and the squilloids (S1–S4) (Fig. 1a). Clades L1 and S2 both contained over 10 larval sequences collected over multiple years. The remaining seven larval sequences did not cluster with any other sequences. Counting included reference sequences and without splitting sequences published as the same species into multiple putative species, our analysis gives a total of 44 putative species based on genetic data alone.

PTP analysis resulted in 62 putative species delimitations with varying support, 14 of which contained a single larval sequence with no reference sequences. Several species which contained multiple reference sequences were divided into two, three, or even four putative species (Table 1). Clades L1–L3 and S1–S4 which contained no adult reference sequences were supported by the PTP analysis while clades G1 and L4 were divided into two putative species each containing a single sequence.

**Table 1:** Minimum and maximum percent divergence within each of our OTU designations.

Species	Larvae	Previously identified	Min. % divergence	Max. % divergence
Gonodactyloidea				
*Chorisquilla hystrix*	**2**	**1**	**0.31**	**0.94**
*Chorisquilla tweediei*	**6**	**1**	**0.26**	**1.77**
*Gonodactylaceus falcatus*	**68**	**6**	**0**	**5.59**
sp. 1	68	4		
sp. 2		2		
*Gonodactylellus affinis*	**6**	**3**	**0**	**3.98**
sp. 1	6	2		
sp. 2		1		
*Gonodactylus childi*	**1**	**5**	**0.17**	**4.51**
sp. 1	1	2		
sp. 2		1		
sp. 3		1		
sp. 4		1		
*Gonodactylus chiragra*	**1**	**2**	**0.47**	**11.27**
sp. 1	1	1		
sp. 2		1		
*Gonodactylus smithii*	**7**	**4**	**0**	**19.58**
sp. 1	6	3		
sp. 2		1		
sp. 3	1			
*Haptosquilla glyptocercus*	**6**	**2**	**0.16**	**6.71**
sp. 1	5	1		
sp. 2		1		
sp. 3	1			
*Haptosquilla trisponosa*	**14**	**1**	**0**	**1.31**
*Odontodactylus cultrifer*	**3**	**2**	**0.26**	**5.39**
sp. 1	1	1		
sp. 2		1		
sp. 3	2			
*Pseudosquilla ciliata*	**3**	**3**	**0**	**2.2**
Clade G1	**2**		**-**	**0**
sp. 1	1			
sp. 2	1			
Lysiosquilloidea				
*Alachosquilla vicina*	**19**	**2**	**0**	**1.58**
*Lysiosquillina maculata*	**8**	**3**	**0**	**2.12**
*Pullosquilla litoralis*	**6**	**3**	**0.26**	**1.44**
*Pullosquilla thomassini*	**58**	**10**	**0**	**2.77**
Clade L1	**18**		**0**	**2.24**
Clade L2	**3**		**0**	**0.66**
Clade L3	**3**		**0.13**	**0.52**
Clade L4	**2**		**-**	**2.12**
sp. 1	1			
sp. 2	1			
Squilloidea				
*Alima orientalis*	**5**	**3**	**0**	**2.86**
*Alima pacifica*	**38**	**11**	**0**	**3.54**
*Busquilla plantei*	**1**	**1**	**-**	**3.27**
sp. 1		1		
sp. 2	1			
Clade S1	**2**		**-**	**1.52**
Clade S2	**11**		**0**	**1.16**
Clade S3	**3**		**0.26**	**0.74**
Clade S4	**3**		**0.1**	**1.15**

Secondary rows show where our designations disagree with PTP analysis. In those cases, putative species are designated by number. ABGD results were not included as they matched our OTU designations in almost every case. The number of sequences generated from larvae found at Lizard Island is shown in the first column and the number of sequences which were either identified by the authors or downloaded from the NCBI database is shown in the second column. Percent difference for clades with only two sequences is given in the maximum percent divergence column.

ABGD analysis resulted in a recursive partition of 44 distinct clusters for both JC69 and K80 with a prior intraspecific divergence value 0.03 and above. These partitions matched our designations when using a 5% cut off for all clades with the exceptions of *G. chiragra* (containing 3 sequences) which was split into two clusters with a published sequence in each, a division which matched PTP results, and *G. smithii* in which one published sequence (DQ440603) was split into a new cluster along with a larval sequence (MK397448). In addition, ABGD analysis grouped *H. trispinosa* and *H. glyptocercus* together and combined clade L4 (containing 2 sequences) with *P. litoralis*.

The number of putative species designated by PTP analysis did not perfectly match the clusters found by ABGD at any prior value. A prior distance of 0.0024 resulted in a recursive partition of 71 distinct clusters while increasing the prior distance to 0.0029 resulted in a recursive partition of 55 clusters for both JC69 and K80.

There is genetic structure within the identified stomatopod OTUs at many levels. The *G. falcatus* clade, for example, contains a majority of sequences within 3% divergence of each other. It also contains two sequences, from species which are now synonymous with *G. falcatus*, (e.g. *Gonodactylus mutatus* and *Gonodactylus aloha*), which are over 98% similar to each other yet only about 95% similar to all other *G. falcatus* sequences. This pattern is amplified in small clades such as *Gonodactylus smithii* and *Gonodactylus chiragra*. These clades have maximum percent divergences which are much higher than 5% caused by published sequences which are divergent from each other (Table 1). This creates two separate clades, supported by PTP analysis, both with sequences published as the same species.

For four species of stomatopod found at Lizard Island, *Gonodactylellus affinis*, *Lysiosquillina maculata*, *Pseudosquillana richeri* and *Pullosquilla litoralis*, DNA has only been extracted from individuals captured as larvae. Of these species all but *P. richeri* have been morphologically identified from the area as adults and all have published COI sequences available from other regions. Though only larval specimens of *G. affinis* and *P. litoralis* were collected from Lizard Island, we are confident in their identification since these sequences exhibit under 4% sequence divergence to adult references captured elsewhere. *L. maculata* larvae were allowed to grow to the juvenile stage when adult characteristics appear and were identified morphologically before preservation. One larval sequence (MT179665) matched most closely to a species which was not previously collected at Lizard Island, *Busquilla plantei.*

**Figure 2:**
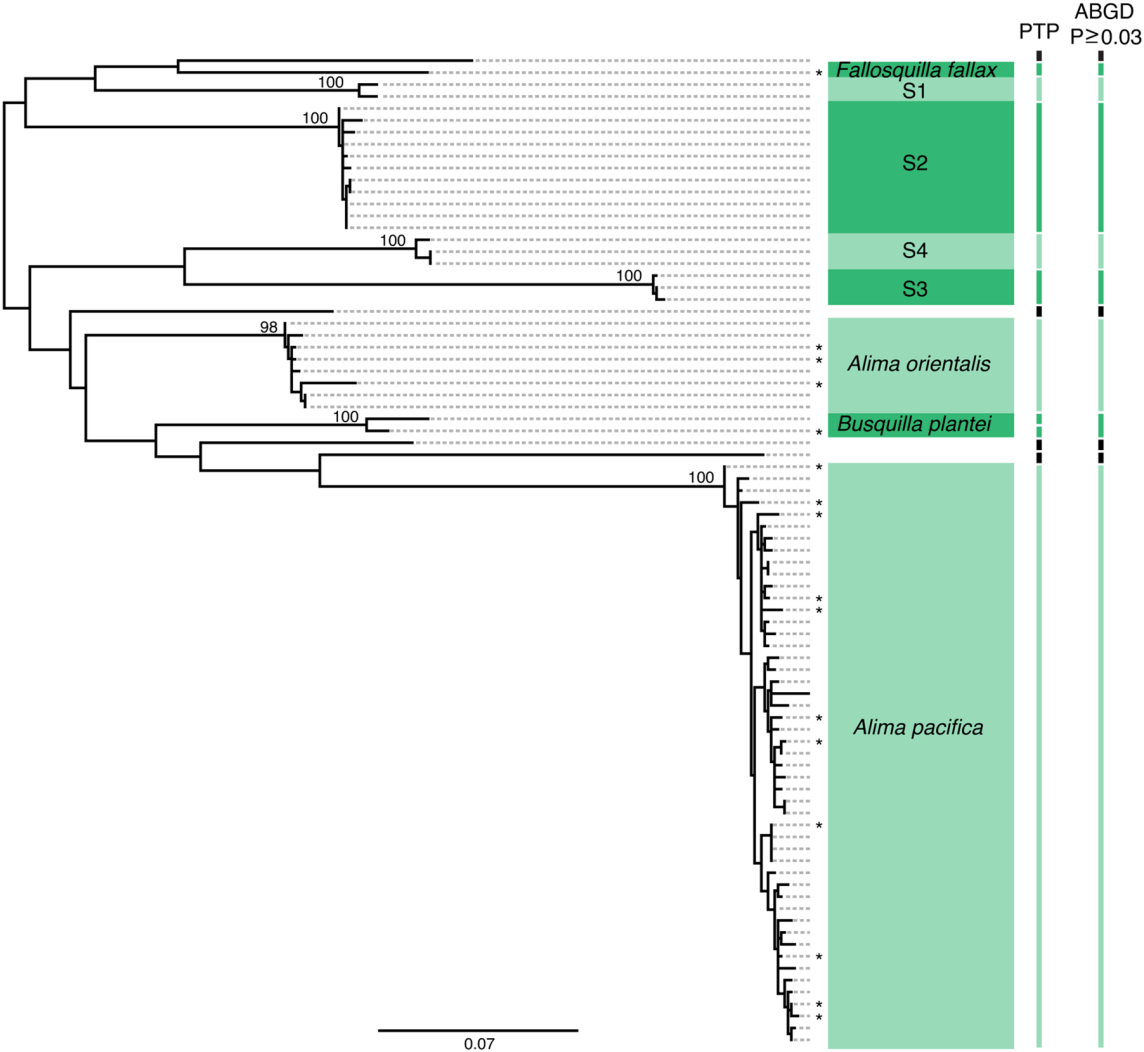
COI gene tree of species in the superfamily Squilloidea found at Lizard Island. (*) symbols represent reference sequences. Each clade is labeled based on reference sequences except for clades S1–S4, which contained only larval sequences. Bars to the right of the tree show the results of PTP and ABGD analysis with each bar indicating a putative species or species cluster. White spaces and black bars indicate single unknown larval sequences. Node values show bootstrap support for each clade.

**Figure 3:**
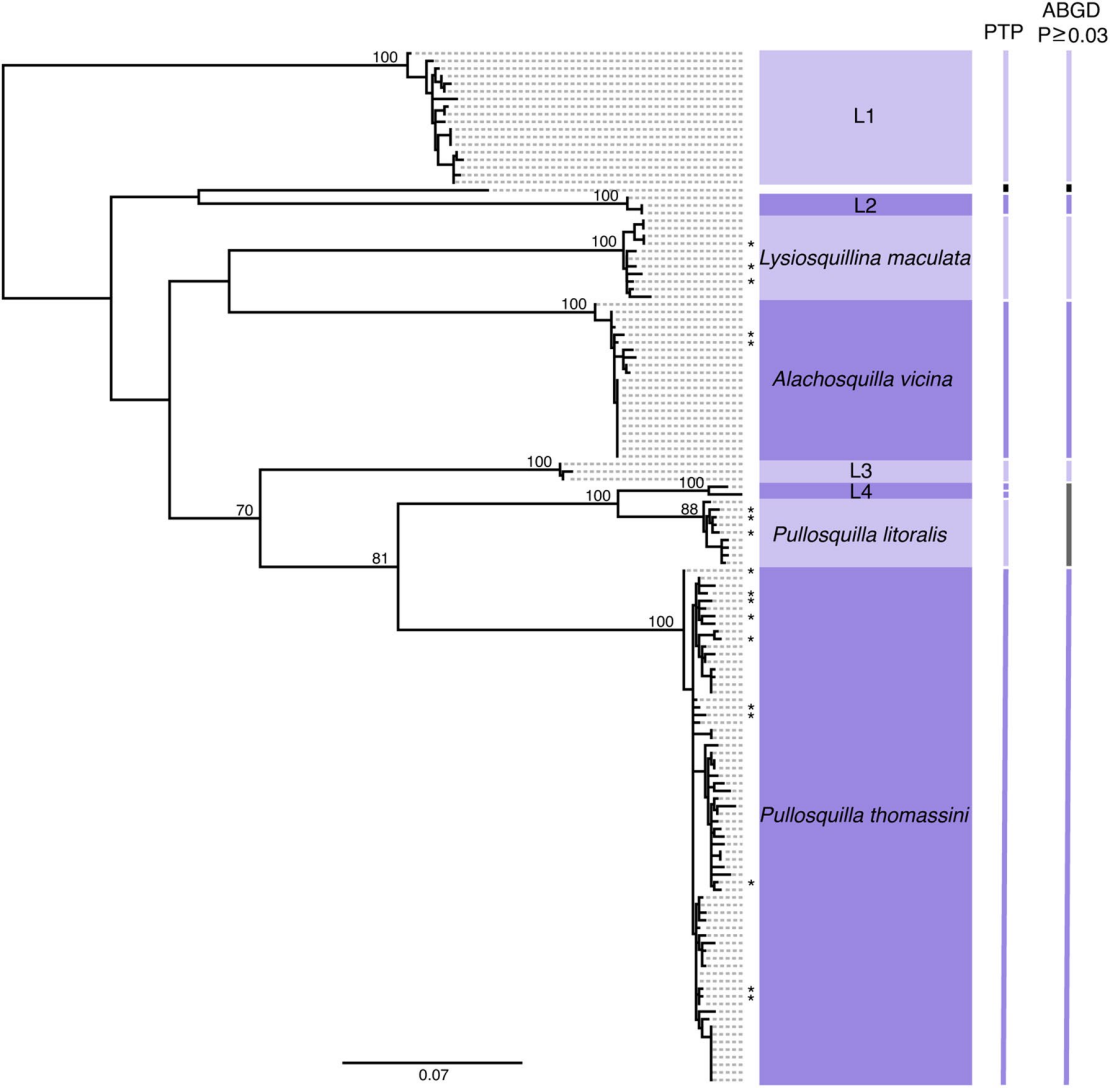
COI gene tree of species in the superfamily Lysiosquilloidea found at Lizard Island. (*) symbols represent reference sequences. Each clade is labeled based on reference sequences except for clades L1–L4, which contained only larval sequences. Bars to the right of the tree show the results of PTP and ABGD analysis with each bar indicating a putative species or species cluster. White spaces and black bars indicate single unknown larval sequences. Dark grey bars indicate species clusters which contain more than one of our previously designated OTUs. Node values show bootstrap support for each clade.

Though a few of the recorded Lizard Island species based on adult collections lack genetic information, the majority of previously identified species from the region can be accounted for with COI barcoding data from the greater Indo-Pacific region. Exceptions where adult species were morphologically identified from Lizard Island but lacked COI data at time of publication include two gonodactyloid (*Gonodactylellus kume* and *Gonodactylellus micronesicus*) and three lysiosquilloid (*Acanthosquilla derijardi*, *Lysiosquilla suthersi* and *Lysiosquillina sulcata*) species. Overall the lysiosquilloids and squilloids appear to be less well sampled than the Lizard Island gonodactyloids as evidenced by the fact that nearly half of our designated OTUs from these superfamilies did not contain a published COI sequence from an identified adult (Figs. 2, 3, see Supplementary Figs. S2 and S3 for individual sequence information).

## Discussion

Single sequence OTUs were disregarded in this study due to the ambiguity associated with them^34^ and a desire for conservative estimations of species richness. Species from which only adult sequences exist are all represented either by a single sequence collected at the Lizard Island reef platform (six species) or by a morphologically identified sample for which COI data was collected elsewhere (four species). This may indicate that exhaustive and consistent sampling methods are needed to accurately account for rare stomatopod species. Additionally, more sampling may be needed to determine the validity of species identifications based on morphology alone. Adult collection records were compiled steadily over the last 30 years or more [12, R. Caldwell, personal communication]. Though larval sampling for this study took place in only a seven-year time span, the species sampling results recapitulate adult records for the most part and indicate that species abundance may be greater than shown by adult sampling alone.

Because sampling effort and exact time of sampling were not consistent between years, it is possible that the overabundance of some sampled larvae (*G. falcatus*, *H. trispinosa*, *A. vicina*, and *P. thomassini*) and the absence of others is a result of variability in seasonality^35^ or lunar periodicity^20,36^ of larval release. In addition, during the course of this study our sampling site was hit by two successive cyclones in 2014 and 2015 which may have contributed to unusual dispersal patterns of pelagic larvae and the advection of rare species into our study area^37^. The inclusion of rare samples may have affected our ABGD analysis, causing some clusters which contained fewer than five sequences to be inappropriately combined or separated.

The similarity between a larval stomatopod sequence (HM138775) and a published *B. plantei* sequence (MT179665) under our OTU estimation method was unexpected, as to our knowledge adult *B. plantei* have not been collected from the Lizard Island reef platform. This species has however been identified from other collections in Eastern Australia^12^ and the existence of an adult population at Lizard Island is possible. Though information on the pelagic larval duration of *B. plantei* is not available, estimates of 3.5 to 6 weeks for other stomatopod species^14,38,39^ leave open the possibility that this larva may have originated elsewhere. Further studies on stomatopod larval dispersal in Eastern Australia would be necessary to expand on this idea. PTP analysis did not support the designation of these two sequences as a single putative species. Instead each sequence was assigned as an individual putative species.

Differences in ability to identify larvae to species varied among superfamilies, and typically followed a pattern dictated by which species are easiest to catch as adults. Gonodactyloids tend to live in coral reef and rubble habitats that are relatively shallow, making them easy to access and subsequently sample^12^. Gonodactyloids also tend to be more brightly colored than lysiosquilloids and squilloids and leave their burrows to hunt^40^, which makes them easier to both spot and capture. The few pseudosquilloid species present at Lizard Island also tend to live in coral rubble^41^ and, in the case of *Pseudosquilla ciliata*, can be very brightly colored and as easy to capture as the other gonodactyloids^12^. Squilloids and lysiosquilloids on the other hand tend to live in sandy or silty burrows dug up to a meter in depth and located in harder to access areas such as mangrove groves or deep water (> 15 m depth)^12^. While many gonodactyloids, can be collected with a simple hand net or by bringing rubble to the surface, collecting lysiosquilloids and squilloids often requires specialized equipment to remove sand or lure animals from their burrows. In areas where mantis shrimp are consumed as food, trawling has proven to be an effective capture strategy^15,35,42^ but is inappropriately destructive for a study such as this in a marine protection zone.

The categorization of OTUs by a specific percent difference cutoff among COI genes presents an interesting problem when we consider references that are initially identified by their morphology. A 5% sequence divergence is highly dependent on accurate published data associated with adult reference sequences^43^. The families that were most affected by this issue were those that were seemingly well sampled, with the gonodactyloids showing the most variation in percent difference within species.

The amount of COI variation within the *G. falcatus* clade (up to 5.59%) presented in this study is a direct result of high levels of divergence in published sequences. In particular two sequences, AF205232 (published as *G. mutatus*) and AF205251 (published as *G. aloha*), were synonymized by current systematics as *G. falcatus*. These two sequences closely match each other and are within 5% divergence of some other published *G. falcatus* sequences, however they are divergent from the majority of published sequences and fall out as their own clade within *G. falcatus*. PTP analysis assigned these two sequences to a separate putative species than all other published *G. falcatus* sequences (Supplementary Table S2).

In *Gonodactylus smithii* this problem is even more extreme due to a published sequence (DQ440603) that closely matches a larval sequence but is almost 20% different from the remainder of the *G. smithii* clade, which contains three published reference sequences. Percent divergences above 5% in the *Gonodactylus chiragra*, *Haptosquilla glyptocercus*, and *Odontodactylus cultrifer* clades are all caused by a single published sequence (AF205250, AF205239, and KM982435, respectively) that is highly divergent from other sequences in the clade. In all of the above cases species were split into at least two putative species by PTP analysis. While these divergence levels could be an artifact of sampling a single gene which may vary in sequence over the geographic range of a species^34,43^, they are still rare within a species^44^ and may represent misidentified samples or geographically distant species within a species complex^45–47^. Interestingly this problem does not appear within the comparatively less well sampled squilloids and lysiosquilloids.

**Figure 4:**
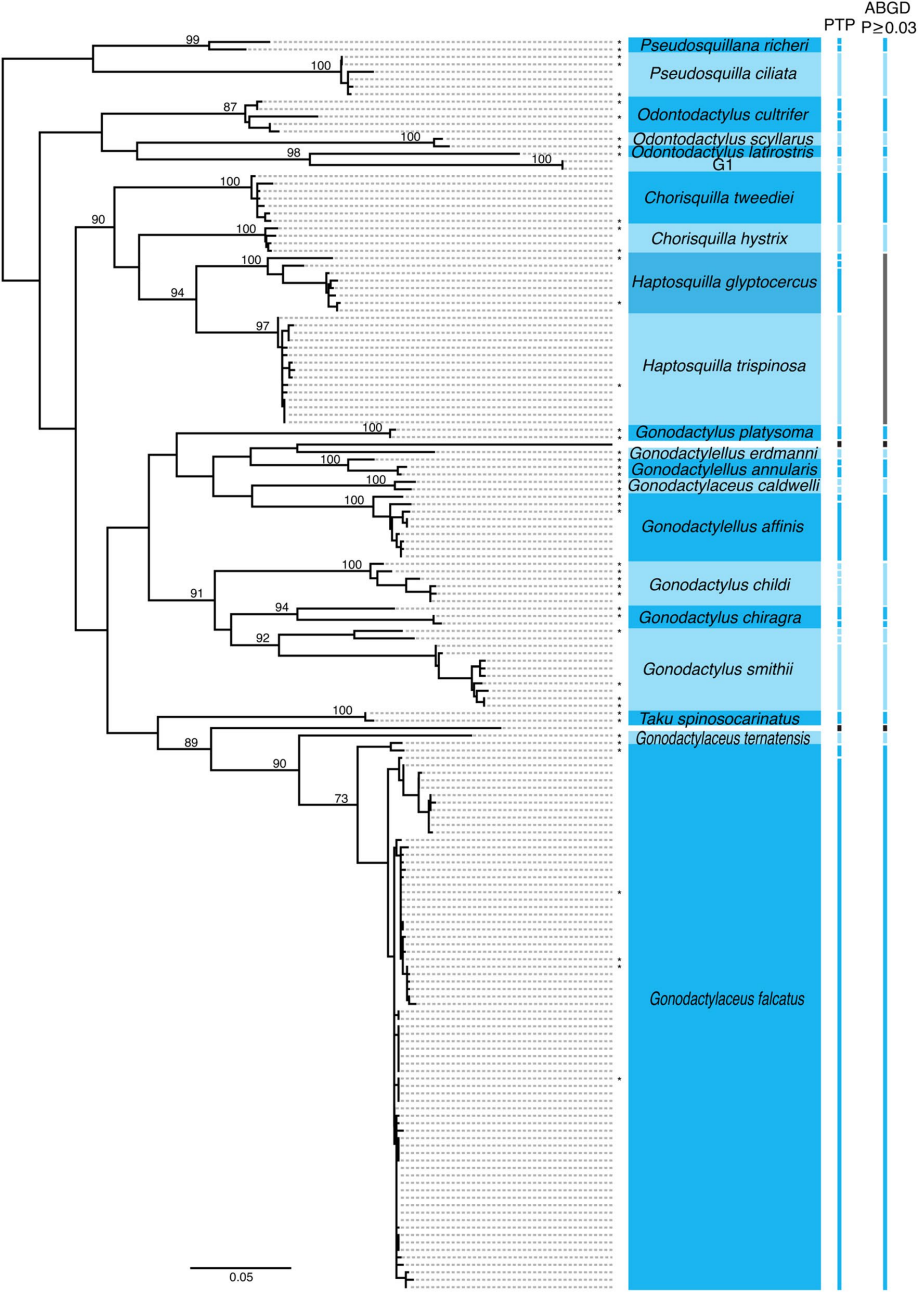
COI gene tree of species in the superfamily Gonodactyloidea found at Lizard Island. (*) symbols represent reference sequences. Each clade is labeled based on reference sequences except for clade G1 which contained two unknown larval sequences. Bars to the right of the tree show the results of PTP and ABGD analysis with each bar indicating a putative species or species cluster. White spaces and black bars indicate single unknown larval sequences. Dark grey bars indicate species clusters which contain more than one of our previously designated OTUs. Node values show bootstrap support for each clade.

Clades present in this study which do not contain a published sequence (G1, L1-4, S1-4) may represent species for which published COI sequences are not available but may also indicate previously unrecorded diversity. It is unlikely that unidentified clade G1 represents sequences from *G. micronesicus* or *G. kume* (gonodactyloid species unrepresented in our study) as it is positioned near members of the *Odontodactylus* genus rather than *Gonodactylellus* (Fig. 4, see Supplementary Fig. S4 for individual sequence information). The G1 clade may represent one or two species present in Lizard Island reef habitats that have yet to be collected as adults. Similarly, it is possible that one of clades L1-4 might be *L. suterthsi* or *L. sulcata* which have previously been identified from Lizard Island based on adult collections. Unfortunately, without comparative sequences from identified adult specimen this cannot be confirmed, leaving at least two lysiosquilloid OTUs to potentially represent species previously undocumented from Lizard Island habitats.

Sampling from the plankton can help to provide an expanded view of species richness and highlight areas which may be missed using traditional sampling methods. Estimates of species richness from adult or larval sampling alone are comparable but less time and energy were needed to obtain larval samples in this study. Furthermore, larval sequences that closely match published larval sequences of unknown identity from other regions provide further information for investigating the connectivity and complex dynamics of larval transport in the ocean. Genetic identification of larval samples also provides a method for tying larval morphological characteristics to a particular species, which ultimately improves identification of samples in the field^20^.

Additional surveys of stomatopods and other marine invertebrates that use unbiased sampling methods for larvae and adults are required to verify the findings of this study. The data presented here provide a baseline for such studies in other systems as well as future work in the Australian Western Pacific Ocean, a location which is undergoing rapid changes as a result of ocean warming. This study demonstrates larval sampling as a viable method for determining species richness in a given location and, with standardized sampling, will be able to generate measures of diversity over time. This method will not be suitable for all taxa and some knowledge of a taxon’s biology is necessary for its application. Only species with a relatively long larval phase can be sampled in this way and information about differences in seasonality of larval release and settlement would be necessary to make sure that all species within a group are being adequately sampled.

Only one mitochondrial marker was used in this study in order to take advantage of already existing data. We acknowledge that increasing evidence for differing speciation rates in mitochondrial and nuclear genomes mean that species delineation based on only one marker is not advisable^48^. Future studies may benefit from the addition of nuclear markers to increase phylogenetic signal and tease apart species complexes.

## Supplementary information

Supplementary file1 (PDF 1514 kb)

Supplementary file 2 (FASTA 294 kb)

## Data Availability

All sequence and alignment data generated or analyzed during this study is available in the GenBank repository (ncbi.nlm.nih.gov/genbank/).
