# Supplementary material for: Using larval barcoding to estimate stomatopod species richness at Lizard Island, Australia for conservation monitoring
Source: Sci Rep. 2020 Jul 3;10:10990. doi: 10.1038/s41598-020-67696-x (PMC7335096; doi:10.1038/s41598-020-67696-x)
Supplement: Supplementary file 1 — Supplementary file1 (PDF 1514 kb) [file 41598_2020_67696_MOESM1_ESM.pdf]

Supplementary Information

Title: Using Larval Barcoding to Estimate Stomatopod Species Richness at Lizard Island, Australia for Conservation Monitoring

Authors: Sitara Palecanda[1§*], Kathryn D. Feller[2§], Megan L. Porter[1]

§ Shared first authorship

* Corresponding author. Email address: spalecan@hawaii.edu

1. Department of Biology, University of Hawai`i at Mānoa, Honolulu HI, USA.
2. Department of Biological Sciences, University of Bristol, Bristol, UK.

Table S1 Complete list of species genetically or morphologically identified from the Lizard Island reef platform. The number of COI sequences collected from each species as adults and as larvae are indicated. Each COI sequence represents one individual. (-) symbols represent species for which COI data is not available from any region but which have been morphologically identified from Lizard Island. Larval sequences were included if they fit within an OTU which contained an adult reference sequence from Lizard Island or elsewhere.

Species	Adult Sequences	Larval Sequences
**GONODACTYLOIDEA**		
Chorisquilla hystrix	1	2
Chorisquilla tweediei	1	6
Gonodactylaceus falcatus	1	70
Gonodactylaceus ternatensis	1	0
Gonodactylellus affinis	0	8
Gonodactylellus annularis	1	0
Gonodactylellus erdmanni	0	0
Gonodactylellus kume	-	-
Gonodactylellus micronesicus	-	-
Gonodactylus caldwelli	0	0
Gonodactylus childi	1	1
Gonodactylus chiragra	1	1
Gonodactylus platysoma	1	0
Gonodactylys smithii	1	7
Haptosquilla glyptocercus	1	6
Haptosquilla trisponosa	1	14
Odontodactylus cultrifer	1	4
Odontodactylus latirostris	1	0
Odontodactylus scyllarus	0	0
Pseudosquilla ciliata	2	3
Pseudosquillana richeri	0	1
Taku spinosocarinatus	1	0
**LYSIOSQUILLOIDEA**		
Alachosquilla vicina	2	19
Acanthosquilla derijardi	-	-
Lysiosquilla suthersi	-	-

Lysiosquillina maculata	0	11
Lysiosquillina sulcata	-	-
Pullosquilla litoralis	0	6
Pullosquilla thomassini	3	65
**SQUILLOIDEA**		
Alima orientalis	1	7
Alima pacifica	1	49
Fallosquilla fallax	1	0

Table S2 GenBank accession numbers for published COI sequences. Reference sequences found on NCBI are in bold. Reference sequences generated for this study are designated by a (*) symbol. Species which were divided into multiple putative species by PTP analysis are shown as such with the full species name written out for the first putative species and subsequent species designated by number. Larval sequences which formed a single sequence OTU are listed by superfamily.

Putative Species	GenBank COI accession numbers
**GONODACTYLOIDEA**	
Chorisquilla hystrix	**HM138777**, MK397464, MK397458
Chorisquilla spinosissima	**AF205254**
Chorisquilla tweediei	**HM138778**, MT188329, MT188330, MT188331, MT188332, MT188333, MT188334
Gonodactylaceus falcatus sp. 1	**HM138786**, **KM982437**, **KM982433**, **AF205230**, MT188224, MT188225, MT188226, MT188227, MT188228, MT188229, MT188230, MT188231, MT188232, MT188233, MT188234, MT188235, MT188236, MT188237, MT188238, MT188239, MT188240, MT188241, MT188242, MT188243, MT188244, MT188245, MT188246, MT188247, MT188248, MT188249, MT188250, MT188251, MT188252, MT188253, MT188254, MT188255, MT188256, MT188257, MT188258, MT188259, MT188260, MT188261, MT188262, MT188263, MT188264, MT188265, MT188266, MT188267, MT188268, MT188269, MT188270, MT188271, MT188272, MT188273, MT188274, MT188275, MT188276, MT188277, MT188278, MT188279, MT188280, MT188281, MT188282, MT188283, MT188284, MT188285, MT188286, MT188287, MT188288, MT188289, MT188290, MT188291
sp. 2	**AF205232**, **AF205251**
Gonodactylaceus ternatensis	**KT001540**
Gonodactylellus affinis sp. 1	**KM982428**, **KM982428**, MK397449, MT188292, MT188293, MT188294, MT188295, MT188296
sp. 2	**AF205228**
Gonodactylellus annularis sp. 1	**DQ440595**, **HM138783**
sp. 2	**AF205226**

Gonodactylellus erdmanni	**GQ260981**
Gonodactylus caldwelli sp. 1	**AF205256**
sp. 2	**DQ440594**
Gonodactylus childi sp. 1	**AF205249**, **HM138784,** MT188297
sp. 2	**AF205246**
sp. 3	**AF205229**
sp. 4	**AF205227**
Gonodactylus chiragra sp. 1	**HM138785,** MT188298
sp. 2	**AF205250**
Gonodactylus platysoma	**AF205237**, **HM138787**
Gonodactylus smithii sp. 1	**AF205233**, **DQ440602**, **HM138788**, MT188299, MT188300, MT188301, MT188302, MT188303, MT188304
sp. 2	**DQ440603**
sp. 3	MK397448
Haptosquilla glyptocercus sp. 1	**HM138789**, MT188305, MT188306, MT188307, MT188308, MT188309
sp. 2	**AF205239**
sp. 3	MT188310
Haptosquilla trispinosa	**HM138790**, MK397457, MT188311, MT188312, MT188313, MT188314, MT188315, MT188316, MT188317, MT188318, MT188319, MT188320, MT188321, MT188322, MT188323
Odontodactylus cultrifer sp. 1	**KM982427**, MT188324
sp. 2	**KM982435**
sp. 3	MK397466, MT188325
Odontodactylus latirostris	**HM138797**
Odontodactylus scyllarus	**AF205234**, **HM138798**
Pseudosquilla ciliata	**AF205245**, **HM138800**, **HM138793**, MT188326, MT188327, MT188328
Pseudosquillana richeri sp. 1	**HM138802**
sp. 2	**KJ828804**
Taku spinosocarinatus	**AF205257**, **HM138811**
Clade G1 sp. 1	MT188335
sp. 2	MT188336
Single Sequence OTUs	MT188337, MT188338
**LYSIOSQUILLOIDEA**	
Alachosquilla vicina	**KM982440**, MT169595*, MK397445, MT169596, MT169597, MT169598, MT169599, MT169600, MT169601, MT169602, MT169603, MT169604, MT169605, MT169606, MT169607, MT169608, MT169609, MT169610, MT169611, MT169612, MT169613
Lysiosquillina maculata	**KM982436**, **KM982432**, **KM982431**, MT169587, MT169588, MT169589, MT169590, MT169591, MT169592, MT169593, MT169594
Pullosquilla litoralis	MT169578*, MT169579*, MT169580*, MT169581, MT169582, MT169583, MT169584, MT169585, MT169586
Pullosquilla thomassini	**HM138803, KJ828810, KJ828809, KJ828808, KJ828807, KJ828806, KJ828805, KM982439, KM982438, KM982425**, MK397442, MK397451, MK397454, MK397461, MK397462, MT153071, MT153072, MT153073, MT153074, MT153202, MT154783, MT154784, MT154785, MT154786, MT154787, MT154788, MT154789, MT154790, MT154791, MT154792,

	MT154793, MT154794, MT154795, MT154796, MT154797, MT169545, MT169546, MT169547, MT169548, MT169549, MT169550, MT169551, MT169552, MT169553, MT169554, MT169555, MT169556, MT169557, MT169558, MT169559, MT169560, MT169561, MT169562, MT169563, MT169564, MT169565, MT169566, MT169567, MT169568, MT169569, MT169570, MT169571, MT169572, MT169573, MT169574, MT169575, MT169576, MT169577
Clade L1	MK397443, MK397444, MK397447, MK397450, MK397452, MK397459, MK397460, MK397465, MK397467, MK397468, MK397469, MK397470, MK397471, MK397472, MT169614, MT169615, MT169616, MT169617
Clade L2	MT169618, MT169619, MT169620
Clade L3	MK397453, KM982429, MT169621
Clade L4 sp. 1	MT169622
sp. 2	MT169623
Single Sequence OTU	MT169624
**SQUILLOIDEA**	
Alima orientalis	**HM138773**, **KF214292**, **KF205337**, MT179622, MT179623, MT179624, MT179625, MT179626
Alima pacifica	**HM138774**, **KM982434**, **KM982424**, **KM982422**, **KM982421**, **KM982420**, **KM982423**, **KF205340**, **KF205341**, **KF205338**, **KF205339**, MT179627, MT179628, MT179629, MT179630, MT179631, MT179632, MT179633, MT179634, MT179635, MT179636, MT179637, MT179638, MT179639, MT179640, MT179641, MT179642, MT179643, MT179644, MT179645, MT179646, MT179647, MT179648, MT179649, MT179650, MT179651, MT179652, MT179653, MT179654, MT179655, MT179656, MT179657, MT179658, MT179659, MT179660, MT179661, MT179662, MT179663, MT179664
Busquilla plantei sp. 1	**HM138775**
sp. 2	MT179665
Fallosquilla fallax	**HM138781**
Clade S1	MK397446, MK397456
Clade S2	KJ828811, MT179666, MT179667, MT179668, MT179669, MT179670, MT179671, MT179672, MT179673, MT179674, MT179675
Clade S3	KM982430, MT179676, MT179677
Clade S4	MT179678, MT179679, MT179680
Single Sequence OTUs	MT179681, MT179682, MT179683, MT179684

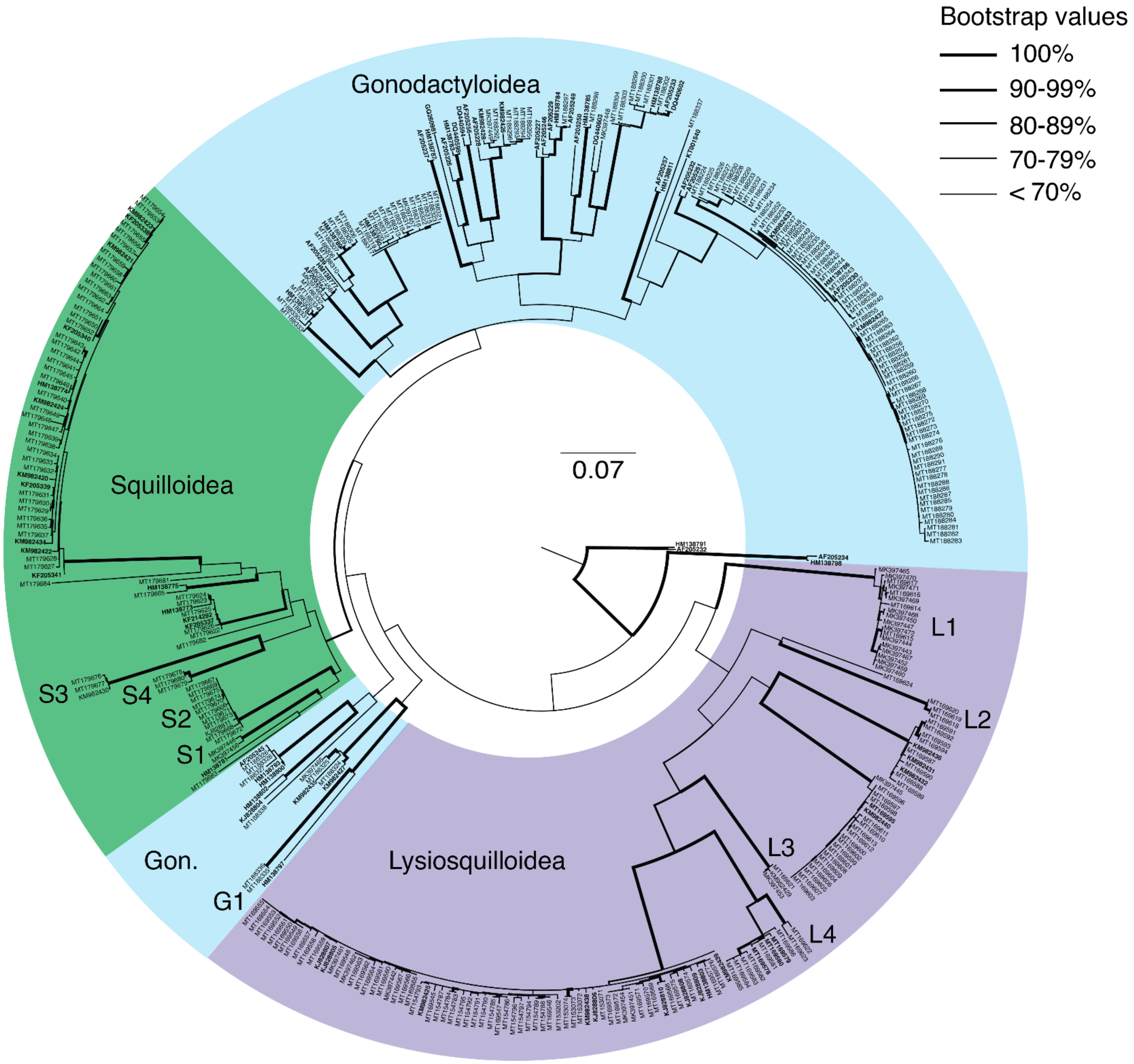

Figure S1 COI gene tree of stomatopod species from Lizard Island. The Gonodactyloidea are rendered paraphyletic. Accession numbers for each sequence are provided. Reference sequences are in bold. Clades containing only larval sequences are labeled by first letter of superfamily (e.g. G1, L1-4, and S1-4); all other clades contain a reference sequence. Branch thickness represents bootstrap value at each node.

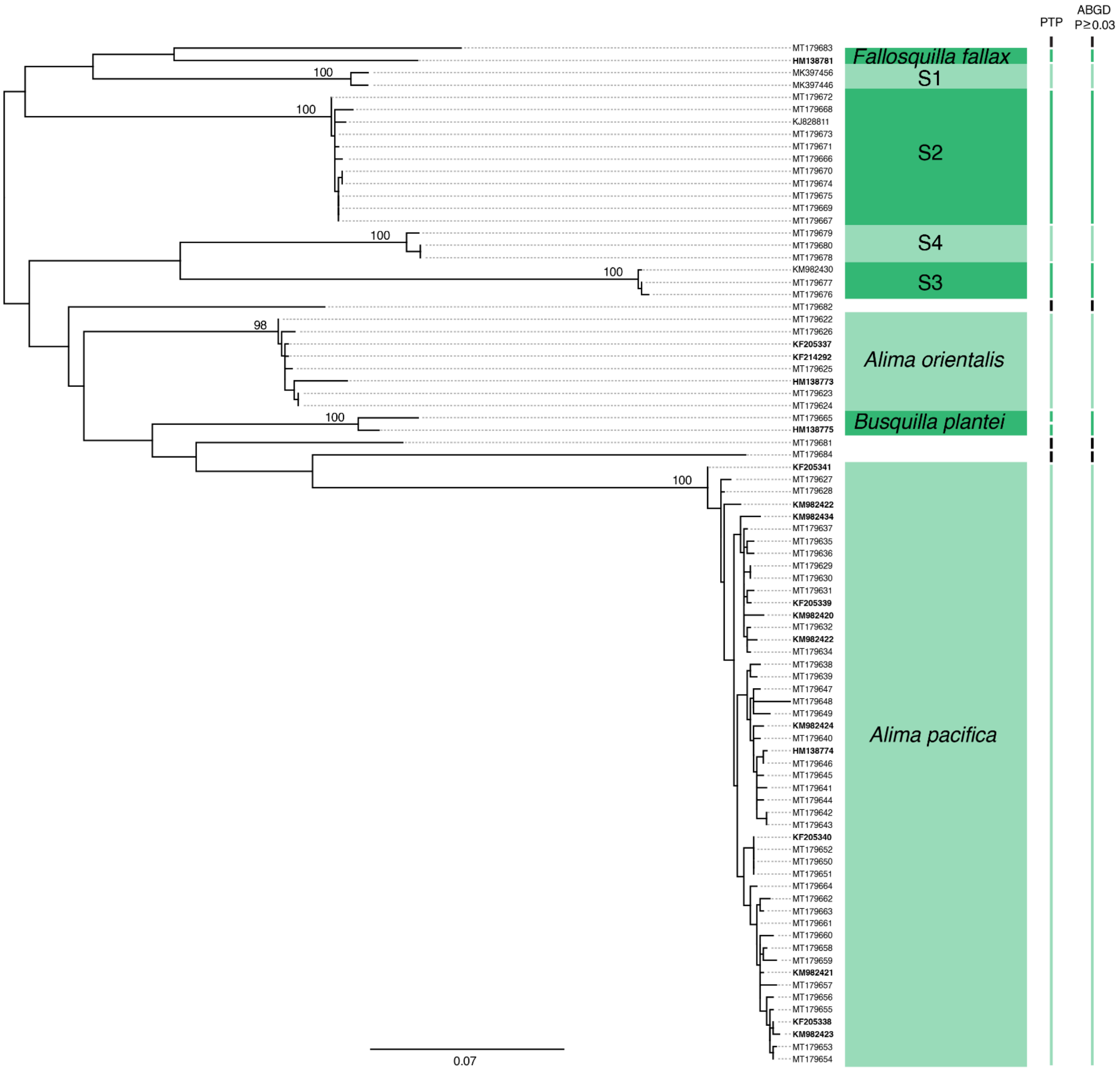

Figure S2 COI gene tree of species in the superfamily Squilloidea found at Lizard Island. Accession numbers for each sequence are provided. Reference sequences are in bold. Each clade is labeled based on reference sequences except for clades S1-S4, which contained only larval sequences. Bars to the right of the tree show the results of PTP and ABGD analysis with each bar indicating a putative species or species cluster. White spaces and black bars indicate single unknown larval sequences. Node values show bootstrap support for each clade.

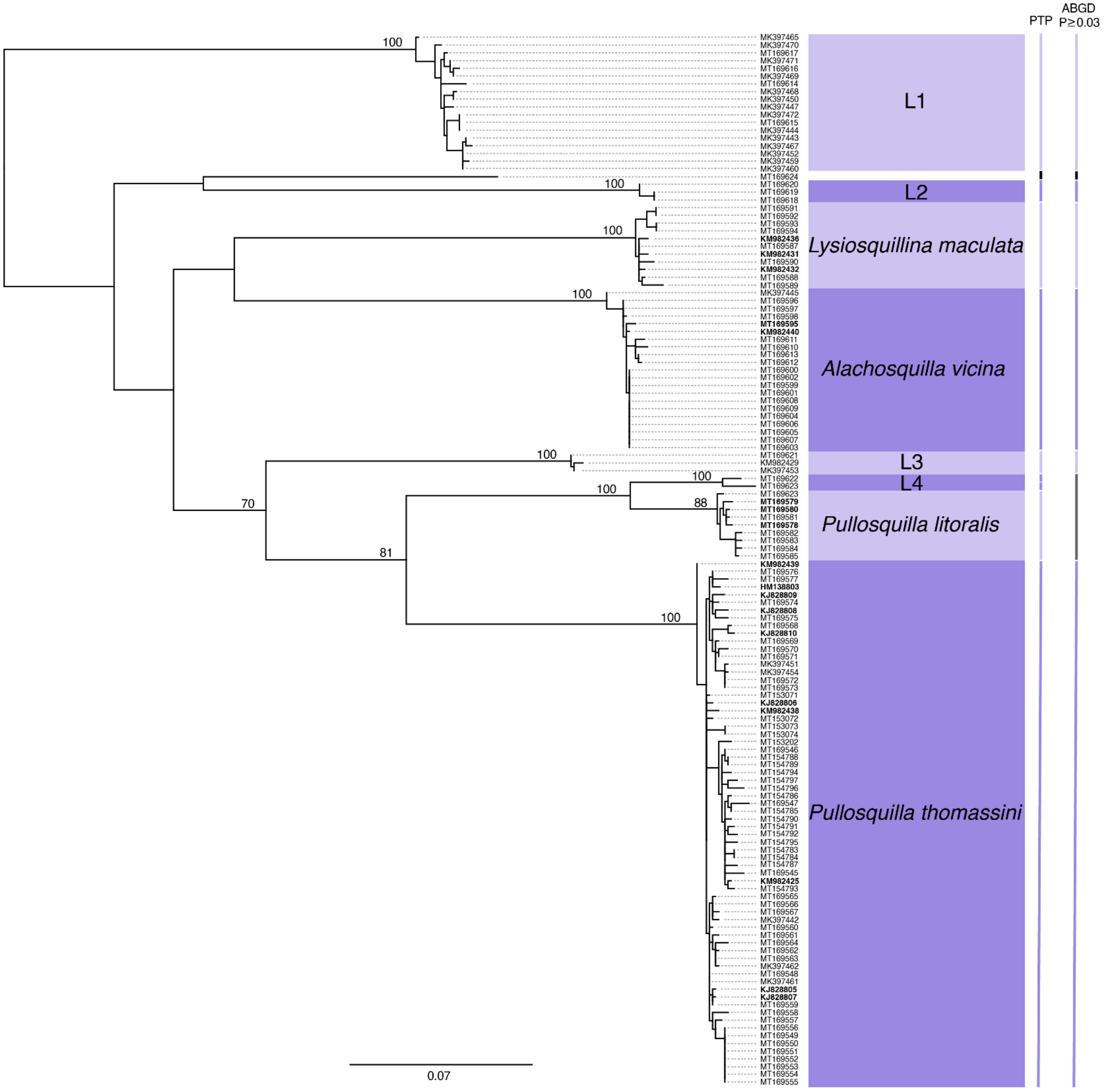

Figure S3 COI gene tree of species in the superfamily Lysiosquilloidea found at Lizard Island. Accession numbers for each sequence are provided. Reference sequences are in bold. Each clade is labeled based on reference sequences except for clades L1 - L4, which contained only larval sequences. Bars to the right of the tree show the results of PTP and ABGD analysis with each bar indicating a putative species or species cluster. White spaces and black bars indicate single unknown larval sequences. Dark grey bars indicate species clusters which contain more than one of our previously designated OTUs. Node values show bootstrap support for each clade

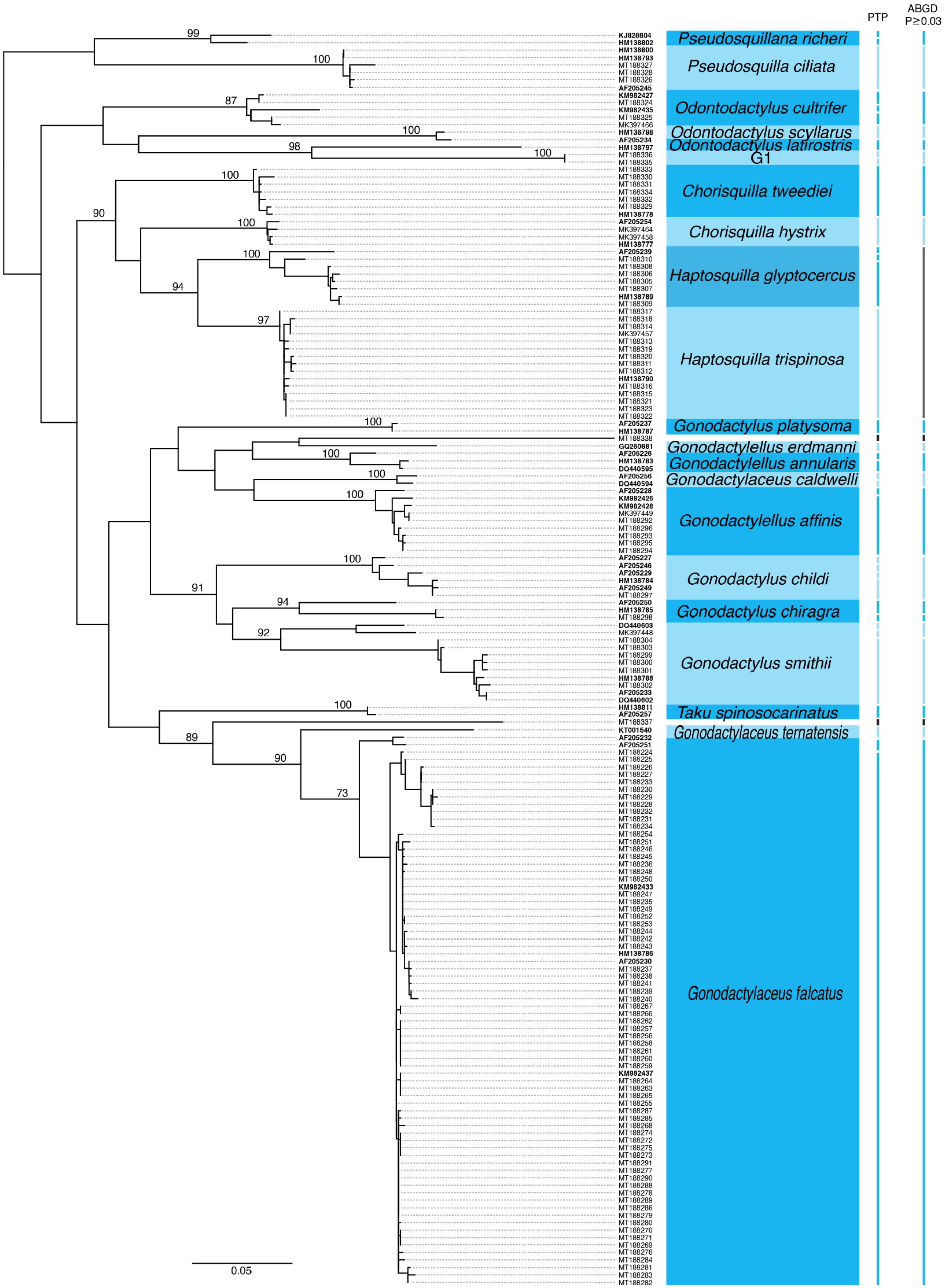
PTP
ABGD
P≥0.03
Pseudosquillana richeri
Pseudosquilla ciliata
Odontodactylus cultrifer
Odontodactylus scyllarus
Odontodactylus latirostris
G1
Chorisquilla tweediei
Chorisquilla hystrix
Haptosquilla glyptocercus
Haptosquilla trispinosa
Gonodactylus platysoma
Gonodactylellus erdmanni
Gonodactylellus annularis
Gonodactylaceus caldwelli
Gonodactylellus affinis
Gonodactylus childi
Gonodactylus chiragra
Gonodactylus smithii
Taku spinosocarinatus
Gonodactylaceus ternatensis
Gonodactylaceus falcatus
0.05
99
100
87
100
98
100
100
90
100
100
94
97
100
100
100
100
91
94
92
100
89
90
73
KJ828804
HM138802
HM138800
HM138793
MT188327
MT188328
MT188326
AF205245
KM982427
MT188324
KM982435
MT188325
MK397466
HM138798
AF205234
HM138797
MT188336
MT188335
MT188333
MT188330
MT188331
MT188334
MT188332
MT188329
HM138778
AF205254
MK397464
MK397458
HM138777
AF205239
MT188310
MT188308
MT188306
MT188305
MT188307
HM138789
MT188309
MT188317
MT188318
MT188314
MK397457
MT188313
MT188319
MT188320
MT188311
MT188312
HM138790
MT188316
MT188315
MT188321
MT188323
MT188322
AF205237
HM138787
MT188338
GQ260981
AF205226
HM138783
DQ440595
AF205256
DQ440594
AF205228
KM982426
KM982428
MK397449
MT188292
MT188296
MT188293
MT188295
MT188294
AF205227
AF205246
AF205229
HM138784
AF205249
MT188297
AF205250
HM138785
MT188298
DQ440603
MK397448
MT188304
MT188303
MT188299
MT188300
MT188301
HM138788
MT188302
AF205233
DQ440602
HM138811
AF205257
MT188337
KT001540
AF205232
AF205251
MT188224
MT188225
MT188226
MT188227
MT188233
MT188230
MT188229
MT188228
MT188232
MT188231
MT188234
MT188254
MT188251
MT188246
MT188245
MT188236
MT188248
MT188250
KM982433
MT188247
MT188235
MT188249
MT188252
MT188253
MT188244
MT188242
MT188243
HM138786
AF205230
MT188237
MT188238
MT188241
MT188239
MT188240
MT188267
MT188266
MT188262
MT188257
MT188256
MT188258
MT188261
MT188260
MT188259
KM982437
MT188264
MT188263
MT188265
MT188255
MT188287
MT188285
MT188268
MT188274
MT188272
MT188275
MT188273
MT188291
MT188277
MT188290
MT188288
MT188278
MT188289
MT188286
MT188279
MT188280
MT188270
MT188271
MT188269
MT188276
MT188284
MT188281
MT188283
MT188282

Figure S4 COI gene tree of species in the superfamily Gonodactyloidea found at Lizard Island. Accession numbers for each sequence are provided. Reference sequences are in bold. Each clade is labeled based on reference sequences except for clade G1 which contained two unknown larval sequences. Bars to the right of the tree show the results of PTP and ABGD analysis with each bar indicating a putative species or species cluster. White spaces and black bars indicate single unknown larval sequences. Dark grey bars indicate species clusters which contain more than one of our previously designated OTUs. Node values show bootstrap support for each clade.
